# The Opinion as to Quinine in Pneumonia

**Published:** 1881-07

**Authors:** 


The Opinion as to Quinine in Pneumonia.—A writer in the St. Louis *Clinical Record* says: "I think a good many pneumonic patients are killed with quinine. If there is any indication or reason for giving it, I don't know what it is. It disorders the nervous system, impairs digestion. It has no influence in preventing hepatization or hastening resolution. I know a man in my county who has complete amaurosis from taking quinine for pneumonia last winter. His doctor gave him half a bottle in twenty-four hours on the 'vaso-motor,' 'inhibitory,' 'accelerating,'

'depressing,' 'constricting,' 'dilating,' hypothetical theory of the day. But the fashion now is quinine from a stone-bruise to a broken neck. If no more quinine should be used than is really beneficial in disease it wouldn't be worth a dollar a bottle."—*Pacific Med. and Surg. Journal.*

